# Stress-related neuroplasticity and developmental vulnerability in functional neurological disorder: from adverse experience to maladaptive overlearning

**DOI:** 10.3389/fnmol.2026.1816644

**Published:** 2026-06-15

**Authors:** Ioannis Mavroudis, Foivos Petridis, Alin Ciobica, Cǎtǎlina Ionescu, Ahmed Adel Mansour Kamar, Roxana-Oana Cojocariu, Dimitrios Kazis, Diana Gheban, Catalin Morosan, Bogdan Gurzu, Otilia Novac, Bogdan Novac

**Affiliations:** 1 Leeds Teaching Hospitals, NHS Trust, Leeds, United Kingdom; 2 Romanian Academy of Scientists (Academia Oamenilor de Știință din România), Bucharest, Romania; 3 Third Department of Neurology, Aristotle University of Thessaloniki, Thessaloniki, Greece; 4 Department of Biology, Faculty of Biology, “Alexandru Ioan Cuza” University of Iasi, Iasi, Romania; 5 “Ioan Hăulică” Institute, “Apollonia” University of Iași, Iasi, Romania; 6 "Olga Necrasov" Center, Biomedical Research Group, Romanian Academy, Iasi, Romania; 7 Academy of Romanian Scientists, Bucharest, Romania; 8 Doctoral School of Biology, Faculty of Biology, “Alexandru Ioan Cuza” University of Iași, Iasi, Romania; 9 GUPCO – Medical Department, Cairo Office, Cairo, Egypt; 10 Department of Orthopedics and Traumatology, Clinical Recovery Hospital (Recuperare), Iași, Romania; 11 Department of Biological and Morphological Sciences, Faculty of Medicine and Biological Science, Stefan Cel Mare University of Suceava, Suceava, Romania; 12 Faculty of Medicine, Grigore T. Popa University of Medicine and Pharmacy, Iasi, Romania

**Keywords:** early-life trauma and adversity, Functional Neurological Disorder (FND), limbic and sensorimotor circuits, maladaptive learning and bodily predictions, stress-related neuroplasticity

## Abstract

Functional Neurological Disorder (FND) is increasingly understood as a disorder of brain functioning rather than structural damage. While contemporary predictive coding models have provided important insights into the generation of functional symptoms, they offer limited explanations for vulnerability, developmental risk, and the striking association between FND and adverse life experiences. A substantial body of evidence indicates that early-life stress, trauma, and chronic adversity are overrepresented in individuals with FND and are associated with enduring neurobiological alterations in brain systems central to emotional processing, interoception, and motor control. In this paper, we propose that stress-related neuroplasticity constitutes a key developmental substrate that predisposes the brain to maladaptive learning processes, increasing the likelihood that salient bodily states become rigidly encoded and persist as functional symptoms. We review evidence linking trauma exposure to long-lasting changes in limbic, paralimbic, and sensorimotor circuits implicated in FND, including the amygdala, anterior cingulate cortex, insula, orbitofrontal cortex, and supplementary motor areas. We further examine autonomic and neuroendocrine abnormalities and gender-related biological factors that may amplify these effects. We argue that these convergent findings support a model in which FND emerges from excessive, stress-biased neuroplasticity that promotes overlearning of bodily predictions, rather than from a primary failure of belief updating. This framework provides a coherent account of vulnerability, symptom persistence, and heterogeneity in FND, and offers a biologically grounded bridge between developmental risk factors and contemporary computational models of functional symptoms.

## 1 Introduction

Functional Neurological Disorder presents a paradox that has long challenged neurological theory. Patients experience genuine, often disabling neurological symptoms—such as weakness, abnormal movements, sensory loss, or dissociative episodes, yet these symptoms arise in the absence of identifiable structural lesions (Bennett et al., 2021). Over recent decades, advances in cognitive neuroscience have moved the field away from psychogenic or purely psychological explanations toward models grounded in brain-based mechanisms (Miller, 2010; Perez et al., 2021; Mavroudis et al., 2025; Singh, 2024). Among these, predictive coding and active inference frameworks have been particularly influential, reframing functional symptoms as consequences of abnormal perceptual and motor inference rather than conscious or unconscious fabrication (Sterzer et al., 2018).

Despite this conceptual progress, a persistent gap remains between mechanistic models of symptom expression and the well-established clinical observation that FND is strongly associated with adverse life experiences. Epidemiological studies consistently show elevated rates of childhood abuse, neglect, and later-life trauma in individuals with FND, yet these factors are often treated as contextual or epiphenomenal rather than mechanistically central (Berlot et al., 2025). As a result, vulnerability is frequently discussed separately from symptom generation, leading to fragmented explanatory models (Armaș et al., 2025).

In this paper, we argue that stress-related neuroplasticity is not peripheral but foundational to understanding FND. We propose that early and repeated adversity shapes learning dynamics within brain systems responsible for salience detection, interoception, emotion regulation, and motor control. These neuroplastic changes bias the brain toward overly rigid encoding of salient bodily states, increasing the likelihood that transient sensorimotor disturbances become persistently expressed as functional symptoms. This perspective aligns naturally with contemporary computational models while addressing their developmental blind spots.

### 1.1 Adverse life events as risk factors for Functional Neurological Disorder

Systematic reviews and case–control studies consistently demonstrate that childhood maltreatment, encompassing emotional, physical, and sexual abuse, is significantly overrepresented in FND populations compared to neurologically healthy controls (Keynejad et al., 2019; Ludwig et al., 2018; Fobian and Elliott, 2019; Phillips, 2025). Rather than treating these factors as epiphenomenal background, our framework integrates them as neurobiological drivers that lower the threshold for functional symptoms (Bany-Mohammed et al., 2025). These associations are summarized in Table 1.

**Table 1:** Adverse life events and vulnerability to Functional Neurological Disorder.

Factor	Evidence in FND	Neurobiological relevance	References
Childhood emotional abuse	Overrepresented in FND cohorts	Alters limbic reactivity and salience processing	Phillips (2025), Sojka et al. (2018), and Korgaonkar et al. (2026)
Childhood physical abuse	Increased prevalence compared to controls	Affects stress-related neuroplasticity	Pasteuning et al. (2025) and Cross et al. (2017)
Childhood sexual abuse	Strong association in systematic reviews	Sensitizes amygdala and insula	Edwards (2018) and Teicher and Samson (2016)
Repeated early adversity	Cumulative risk factor	Lowers threshold for maladaptive learning	Wadman et al. (2020) and Giovanelli et al. (2020)
Adult traumatic events	Common proximal triggers	Reinforces bodily threat representations	Muldoon et al. (2019)

The relevance of these findings extends beyond epidemiology. Adverse experiences, particularly when occurring during sensitive developmental periods, are known to exert long-lasting effects on brain structure and function (McLaughlin et al., 2014). Rather than producing focal lesions, stress and trauma alter the maturation and plasticity of distributed neural systems involved in threat detection, bodily awareness, and action selection. These effects are subtle, cumulative, and enduring, precisely the kind of changes that could predispose to functional symptoms without producing overt neurological damage (Nguyen and Shetty, 2026; Harnett et al., 2025).

Crucially, FND often emerges following a bodily event, such as pain, injury, illness, or dissociation, that is itself emotionally salient. In individuals with stress-shaped neural systems, such events may be encoded with disproportionate strength, setting the stage for maladaptive persistence (Milano et al., 2023).

### 1.2 Stress-related neuroplasticity in brain networks relevant to FND

Neuroimaging literature confirms that early-life stress fundamentally reshapes key nodes within neural networks responsible for emotional processing, interoception, and motor control (Li et al., 2023; Dannlowski et al., 2012). As detailed alongside their respective imaging modalities in Table 2, these stress-induced alterations compromise structural integrity and functional connectivity across a distributed network (Table 2).

**Table 2:** Brain regions affected by stress-related neuroplasticity and implicated in FND.

Brain region	Stress-related plasticity effects	Relevance to FND	References
Amygdala	Hyperreactivity, volume changes	Emotional salience, threat tagging	Fonzo (2019)
Anterior cingulate cortex	Structural and functional alterations	Action selection, autonomic integration	Bush et al. (2000)
Insula	Altered interoceptive processing	Bodily awareness, symptom salience	Choquette and Khalsa (2025)
Orbitofrontal cortex	Stress-sensitive maturation	Valuation and prediction updating	Girotti et al. (2022)
Hippocampus	Stress-related volume reduction	Contextual modulation of bodily states	Kim et al. (2015)
Supplementary motor area	Limbic–motor coupling	Initiation of functional motor symptoms	Diez et al. (2021)

The amygdala, central to threat detection and emotional salience, shows heightened reactivity following childhood maltreatment. This sensitization persists into adulthood and is associated with exaggerated responses to negative or ambiguous stimuli (Dannlowski et al., 2012). The insula, a key hub for interoceptive awareness, is similarly affected by stress exposure, with alterations that bias attention toward bodily sensations. The anterior cingulate cortex, which integrates emotional salience with motor and autonomic responses, exhibits stress-related structural and functional changes that may alter action selection under uncertainty (Wang et al., 2019).

Within the context of FND, these alterations are particularly relevant. Functional imaging studies in FND demonstrate abnormal engagement of amygdala–motor and insula–sensorimotor networks during symptom expression and emotional processing. Stress-related plasticity may therefore prime these circuits to overrepresent bodily threat and instability, increasing the likelihood that sensorimotor predictions become rigidly encoded (Diez et al., 2019; Günther et al., 2015; Diez et al., 2021).

### 1.3 From stress to maladaptive learning: a neuroplasticity perspective

The enduring impact of trauma on brain function suggests that vulnerability to FND is not simply a matter of psychological coping style or belief formation, but reflects altered learning rules within neural systems. Stress hormones and neuromodulators such as cortisol, noradrenaline, and dopamine directly influence synaptic plasticity, often enhancing learning for emotionally salient stimuli while impairing contextual flexibility (Thomason and Marusak, 2017; Mavroudis et al., 2024).

In practical terms, this means that bodily experiences occurring under conditions of heightened arousal are more likely to be encoded strongly and persistently. Pain, dizziness, dissociation, or motor instability may become “tagged” as highly salient, and repeated attention to these states reinforces their neural representation (Merchie and Gomot, 2023). Over time, this process can lead to excessively specialized encoding of bodily states, reducing the brain’s ability to generalize across contexts (Berens and Bird, 2022).

This form of learning is adaptive in environments characterized by real and persistent threat. However, when applied to bodily sensations in relatively safe contexts, it becomes maladaptive. The nervous system begins to treat transient bodily states as stable predictors, increasing the probability that these states are reactivated even in the absence of ongoing threat (Aboushaar and Serrano, 2024) (Table 3).

**Table 3:** Autonomic and neuroendocrine abnormalities reported in FND.

System	Observed abnormality	Functional implication	References
Sympathetic nervous system	Increased baseline tone	Heightened bodily arousal	Yaribeygi et al. (2017)
Parasympathetic nervous system	Reduced vagal tone	Reduced physiological flexibility	Shaffer and Ginsberg (2017)
Skin conductance	Increased reactivity	Amplified salience of bodily signals	Benedek and Kaernbach (2010)
Startle reflex	Exaggerated responses	Threat-biased sensorimotor coupling	Dreissen et al. (2012)
HPA axis	Dysregulated stress responses	Alters learning and plasticity	Thom et al. (2026)

### 1.4 Emotional processing biases and amygdala sensitization

Evidence from non-clinical populations further supports the link between trauma, emotional processing biases, and vulnerability to FND. Individuals with histories of childhood maltreatment demonstrate automatic biases toward negative emotional stimuli and reduced ability to disengage from threat-related cues (Günther et al., 2015). These biases are accompanied by heightened amygdalar responses, suggesting that emotional salience systems are recalibrated toward increased sensitivity (Lin et al., 2020).

In patients with FND, abnormal amygdala activation has been observed during emotional tasks and symptom provocation, even in the absence of overt emotional distress. This pattern supports the idea that emotional salience is integrated into sensorimotor processing at an implicit level (Sojka et al., 2018). When bodily sensations are repeatedly paired with heightened limbic activation, the resulting neural representations may become disproportionately influential in guiding perception and action (Paulus et al., 2019).

### 1.5 Autonomic dysregulation and bodily salience

Autonomic abnormalities provide an additional pathway through which stress-related neuroplasticity may facilitate FND. Patients with FND exhibit altered autonomic profiles, including heightened sympathetic tone, reduced parasympathetic regulation, and exaggerated physiological responses to emotional or sensory stimuli (Paredes-Echeverri et al., 2022). Altered skin conductance responses and enhanced startle reflexes further indicate increased bodily reactivity (Najström and Jansson, 2007).

Autonomic arousal amplifies interoceptive signals and directs attention inward. Within a stress-shaped nervous system, this amplification may further reinforce learning about bodily instability, increasing the likelihood that these signals dominate perceptual inference. Over time, autonomic dysregulation and maladaptive learning form a self-reinforcing loop that stabilizes functional symptoms (Najström and Jansson, 2007; Greenwood and Garfinkel, 2025).

### 1.6 Oxytocin, epigenetics, and stress sensitivity

Limited and preliminary evidence suggests that oxytocin signaling may modulate vulnerability to FND by influencing emotional salience and stress responsiveness. Certain oxytocin receptor genotypes are associated with heightened amygdala reactivity to negative emotional stimuli, particularly in individuals exposed to childhood maltreatment. Preliminary findings of increased oxytocin receptor gene methylation in FND further support the role of stress-related epigenetic modification (Triana-Del Rio et al., 2022; Apazoglou et al., 2018).

These findings highlight how early adversity can leave molecular “marks” that shape emotional and bodily learning across the lifespan. Rather than causing symptoms directly, such changes may lower the threshold for maladaptive encoding of bodily states, increasing susceptibility to functional symptoms when triggering events occur.

### 1.7 Gender differences and differential vulnerability

FND disproportionately affects women, and gender differences in stress responsivity offer important clues to vulnerability. Women exposed to similar levels of trauma show higher rates of anxiety and affective disorders than men, suggesting sex-specific interactions between stress, neuroplasticity, and emotional regulation (Bradlow et al., 2026). Key gender-related biological mechanisms contributing to vulnerability are outlined in Table 4.

**Table 4:** Gender-related biological factors relevant to FND vulnerability.

Domain	Evidence	Implication for FND	References
HPA axis regulation	Sex differences in stress reactivity	Alters learning rates under stress	Lewis (2016)
Gonadal hormones	Modulate plasticity and emotion	Affects bodily salience encoding	Kelley et al. (2017)
Corticolimbic activation	Greater limbic reactivity in women	Increased emotional weighting of bodily states	Kaiser et al. (2018)
Epigenetic modulation	Sex-specific stress–gene interactions	Stabilises maladaptive predictions	Stoccoro (2025)
Epidemiology	Higher prevalence in women	Supports biological contribution	Wiest (2025)

Biological factors likely contribute to this disparity. Gonadal hormones modulate HPA axis activity, influence synaptic plasticity, and shape limbic reactivity across development. Sex differences in corticolimbic activation patterns and epigenetic responses to stress further modulate learning dynamics (Heck and Handa, 2019). Within the context of FND, these factors may bias women toward heightened salience attribution to bodily signals and increased reinforcement of maladaptive sensorimotor predictions (Rossi et al., 2025).

### 1.8 Implications for understanding Functional Neurological Disorder

Taken together, these findings support a model in which FND arises from the interaction between stress-shaped neuroplasticity and subsequent bodily experiences. Adverse life events do not directly produce functional symptoms; rather, they recalibrate learning systems, alter salience processing, and bias neural plasticity toward overencoding bodily threat (Mavroudis et al., 2024). When later bodily disturbances occur, these systems are primed to encode them rigidly, increasing the likelihood of persistent functional symptoms (Fobian and Elliott, 2019). The proposed mechanistic mapping from stress-related neuroplasticity to maladaptive overlearning is summarized in Table 5. Importantly, this framework should be regarded as a heuristic and hypothetical model rather than an empirically validated mechanistic account. While computational analogies may provide conceptual insight, direct biological validation of overfitting-like processes in FND remains limited.

**Table 5:** Hypothetical conceptual mapping between stress-related neuroplasticity and overfitting-like processes in FND.

Process	Stress-related effect	Overfitting consequence	References
Increased salience	Heightened limbic activation	Narrow training data	Drane et al. (2021)
Repeated attention	Reinforced bodily focus	Parameter inflation	Phillips (2025)
Neuromodulatory gain	Reduced biological regularization	Excessive learning	Drane et al. (2021)
Reduced variability	Rigid network dynamics	Poor generalization	Drane et al. (2021)

This perspective integrates developmental vulnerability with contemporary brain-based models of FND and provides a coherent account of why symptoms often emerge following adversity, why they are resistant to extinction, and why they disproportionately affect women. Importantly, it reframes FND not as a disorder of belief or volition, but as a consequence of stress-biased learning within intact neural systems.

## 2 Conclusion

Functional Neurological Disorder cannot be fully understood without considering the long-term neurobiological consequences of stress and trauma. Stress-related neuroplasticity shapes the very systems responsible for bodily awareness, emotional salience, and action selection, creating a fertile ground for maladaptive learning. By situating FND within this developmental and neuroplastic context, we gain a deeper understanding of vulnerability, symptom persistence, and heterogeneity. This framework complements computational models of functional symptoms and highlights the need for therapeutic approaches that address not only symptom expression but also the underlying learning dynamics shaped by adversity.
